# Disaster Metrics: A Proposed Quantitative Assessment Tool in Complex Humanitarian Emergencies - The Public Health Impact Severity Scale (PHISS)

**DOI:** 10.1371/4f7b4bab0d1a3

**Published:** 2012-08-21

**Authors:** Jamil D. Bayram, Rashid Kysia, Thomas D. Kirsch

## Abstract

Background: Complex Humanitarian Emergencies (CHE) result in rapid degradation of population health and quickly overwhelm indigenous health resources. Numerous governmental, non-governmental, national and international organizations and agencies are involved in the assessment of post-CHE affected populations. To date, there is no entirely quantitative assessment tool conceptualized to measure the public health impact of CHE. Methods: Essential public health parameters in CHE were identified based on the Sphere Project "Minimum Standards", and scoring rubrics were proposed based on the prevailing evidence when applicable. Results: 12 quantitative parameters were identified, representing the four categories of “Minimum Standards for Disaster Response” according to the Sphere Project; health, shelter, food and nutrition, in addition to water and sanitation. The cumulative tool constitutes a quantitative scale, referred to as the Public Health Impact Severity Scale (PHISS), and the score on this scale ranges from a minimum of 0 to a maximum of 100. Conclusion: Quantitative measurement of the public health impact of CHE is germane to accurate assessment, in order to identify the scale and scope of the critical response required for the relief efforts of the affected populations. PHISS is a new conceptual metric tool, proposed to add an objective quantitative dimension to the post-CHE assessment arsenal. PHISS has not yet been validated, and studies are needed with prospective data collection to test its validity, feasibility and reliability. Citation: Bayram JD, Kysia R, Kirsch TD. Disaster Metrics: A Proposed Quantitative Assessment Tool in Complex Humanitarian Emergencies – The Public Health Impact Severity Scale (PHISS). PLOS Currents Disasters. 2012 Aug 21. doi: 10.1371/4f7b4bab0d1a3.

## Background

**Port-Au-Prince, Haiti 2010:**
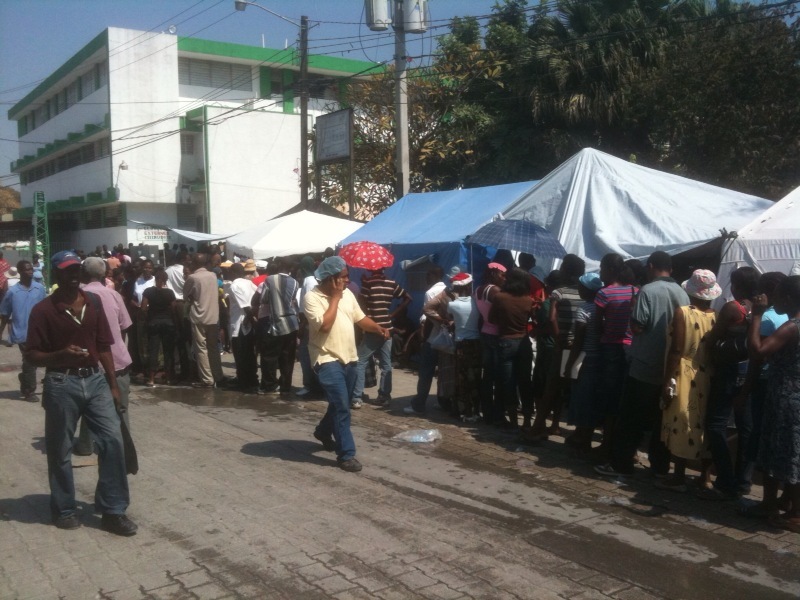

Complex Humanitarian Emergencies (CHE) constitute a multifaceted global public health threat. Contextually, there are multiple interpretations of the term CHE. The United Nations (UN) Inter-Agency Steering Committee defines CHE as: “A humanitarian crisis in a country, region or society where there is a total or significant breakdown of authority resulting from internal or external conflict and which requires an international response that goes beyond the mandate or capacity of any single agency and/or the ongoing UN country program. Common characteristics include: civilian casualties, and populations besieged or displaced; serious political or conflict-related impediments to delivery of assistance; inability of people to pursue normal social, political or economic activities; high security risks for relief workers; and international and cross-border operations affected by political differences.”^1^ CHE can result from natural or man-made events, including armed conflict. From 1990 to 1999, 38 major conflicts with catastrophic public health consequences occurred causing the displacement of millions of people; 70% of which were children and adolescents.^2^

The Sphere Project, launched in 1997 by a group of humanitarian non-governmental organizations and the Red Cross and Red Crescent movement, proposed a set of public health “Minimum Standards for Disaster Response” that need to be monitored and addressed in various phases of disaster response. The four categories of minimum standards are health, food and nutrition, shelter, water and sanitation. The Sphere Project is now in its third edition (2011), and is considered the foundation of public health response in CHE.^3^

To evaluate the public health impact of CHE, in order to provide guidance for disaster relief efforts, organizations and agencies have adopted a myriad of assessment tools. Currently, individual agencies conduct separate assessments utilizing a multiplicity of methods for data-gathering and reporting.^4^ ^5^ ^6^ ^7^ For example, Médecins Sans Frontières (Doctors Without Borders) conducts cross-sectional sample surveys, to obtain information on the most frequent diseases, mortality rates, nutritional status, and on the vaccination status of the population affected.^4^ More recently, the Inter-Agency Standing Committee (IASC) proposed an Initial Rapid Assessment Tool (IRA) which is a mixed (qualitative and quantitative) tool.^6^ ^7^ None of the tools proposed, however, put forth an entirely quantitative public health severity scale based on The Sphere Project "Minimum Standards", that would complement qualitative analysis for the impact of the CHE. Following the 2010 Haiti earthquake, the initial assessment, named the Multi-Cluster Rapid Initial Situational Assessment for Haiti, took over 25 days after the earthquake to be completed, and over 40 days to be analyzed and distributed.^8^ The assessment methods and results in Haiti were criticized by the Centers for Disease Control and Prevention.^9^ A quantitative assessment tool can be used by emergency response planners and policy makers as a source of objective measures on each of the four categories of the "Minimum Standards", in addition to an overall measure of the “big picture” related to the public health impact of CHE. This would help quantify the magnitude of urgent needs, improving the ability to respond expeditiously and appropriately, to measure progress across time, and to compare various complex emergencies.

## Objective

The objective of this study is to propose a conceptual quantitative assessment tool to measure the severity of the public health impact resulting from Complex Humanitarian Emergencies (CHE), based on The Sphere Project "Minimum Standards". Testing the feasibility and validity of the proposed tool is not the objective of this manuscript. Acknowledging the importance of qualitative analysis in conjunction with quantitative data, the proposed tool, henceforth referred to as the Public Health Impact Severity Scale (PHISS), complements and builds upon existing tools and qualitative analyses by providing an objective, quantitative and cumulative measures.

## Methods

Based on the four “Minimum Standards” listed in The Sphere Project 2011 (i.e. health, food and nutrition, shelter, water and sanitation), the authors identified relevant essential public health indicators in CHE. By consensus, the final group of indicators (parameters) was selected, and the scoring rubric for each parameter was stochastically assigned. Whenever applicable, already existing standardized parameters and scores were adopted. The baseline and ceiling scoring values for each parameter, based on criteria available in the literature. Various examples from actual retrospective CHE were provided illustrating how to utilize the scoring rubric for each parameter. Inclusion criteria for each parameter were quantifiability, and being a public health indicator based on The Sphere Project.

## Results

Twelve parameters were identified, representing the four categories of “Minimum Standards” according to The Sphere Project 2011; namely health, shelter, food and nutrition, in addition to water and sanitation. These parameters were: number of excess deaths, number of under-5 excess deaths, number of cases with acute communicable diseases, number of cases with injuries due to the CHE, levels of healthcare services, number of young children with acute malnutrition, level of healthcare services, number of displaced persons, number of persons with inadequate living space, water quantity, water quality, level of sanitation facilities, and gender-based violence. The cumulative scale (PHISS) is quantitative, with a score ranging from a minimum of 0 to a maximum of 100. Below is a detailed description of each parameter selected, the basis for parameter selection, and their suggested scoring rubric, with representative examples.

**1. Number of Excess Deaths (Excess Mortality):** Excess mortality is defined as the mortality attributable to the crisis conditions, above and beyond deaths that would have occurred in normal conditions, and is expressed as total number of excess deaths.^10^ It is calculated by multiplying the difference between the observed Crude Mortality rate (CMR) and the baseline CMR, by the number of the population affected during a certain period of time [(observed CMR – baseline non-crisis CMR) x population at risk x period of time]. Since this tool is utilized in the context of assessment, the period of time need to be clarified, for example it can be set from the onset of the CHE until the assessment is conducted, or during the last month. The reason excess mortality is used as the mortality parameter, rather than the CMR, is to depict the overall mortality burden attributed to the CHE. rates can be very high but the affected population maybe very small. Excess mortality incorporates the mortality rate (CMR) which is the single most important indicator of serious stress (e.g. illness and malnutrition) on affected populations, and has long been a prime public health indicator of the severity of CHE. Of note, both the United Nations High Commissioner for Refugees (UNHCR) and Médecins Sans Frontières (MSF)-Epicentre have determined that the emergency benchmark for CMR is 1 per 10,000 per day.^11^ ^12^

The floor of the scoring rubric for this parameter was chosen to be 10 deaths. This number stems from the criteria adopted by the Centre for Research on the Epidemiology of Disasters (CRED); a collaborative initiative established in 1988 by the WHO and the Belgian government that has been maintaining a database on international disasters named EM-DAT.^13^ For a “disaster” to be entered into the database, at least one of the following criteria must be fulfilled: Ten (10) or more people reported killed, one hundred (100) or more people reported affected, declaration of a state of emergency, or call for international assistance. The 1970 Bangladesh meteorological disaster (cyclone) killed more than 300,000 persons.^13^ According to the CRED database (EM-DAT), it constitutes a CHE that caused the highest number of deaths recorded from 1900 to 2011.^13^ Based on this data, the ceiling of the scoring rubric is therefore chosen to be 300,000. Below is a table showing multiple examples (some CHE and others not), with their corresponding scores.

**Table 1. Total Number of Excess Deaths:** 

**Total number of excess** **deaths**	**Severity** **score**	**Country, year, type**	**Number of** **deaths**
<10	0	None recorded by EM-DAT	N/A
10-99	1	Greece, 2007, forest fire	67
100-999	2	United States, 1947, explosion	561
1,000-4,999	3	Papua New Guinea, 1951, volcanic eruption	3,000
5,000-9,999	4	Germany 2003, heatwave	9,355
10,000-19,999	5	Soviet Union, 1949, landslide	12,000
20,000-49,999	6	India, 1942, tropical storm	40,000
50,000-99,999	7	Pakistan, 2005, earthquake	73,338
100,000-199,999	8	Japan, 1945, atomic bomb	105,000
200,000-299,999	9	Haiti, 2010, earthquake	222,570
Greater than or equal to 300,000	10	Bangladesh, 1970, tropical cyclone	300,000

**2. Number of Under-5 Excess Deaths:** Under-five (year old) excess mortality rate (U5MR) is calculated in the same way as the parameter above, using the difference between the observed and baseline under-5 mortality rates, and multiplying the result by the estimated affected population of that age group per the time period since the onset of CHE. The reason to include this parameter, having already included the one above (total number of excess deaths), is because it better reflects the most vulnerable group, since the under-5 mortality rate is an early sensitive indicator of the mortality impact of CHE. Both UNHCR and MSF have determined that the emergency benchmark for U5MR is 2 per 10,000 per day.^11^ ^12^

**Table 2. Number of Under-5 Excess Deaths:** 

Number of under-5 excess deaths	Severity score
<10	0
10-99	1
100-999	2
1,000-4,999	3
5,000-9,999	4
10,000-19,999	5
20,000-29,999	6
30,000-39,999	7
40,000-49,999	8
50,000-59,999	9
Greater than or equal to 60,000	10

The floor of the scoring rubric for this parameter was chosen to be 10 deaths, based on CRED criteria. The remainder of this scoring rubric, beyond the baseline, is stochastic. For illustration purposes, it was estimated that 46,900 excess deaths among young Iraqi children (under-5 years old) occurred from January 1991 through August 1991, due to the Gulf War and subsequent sanctions.^14^ Based on this parameter, this particular CHE would be assigned a score of 8.

**3. Number of Cases with Acute Communicable Diseases:** Following a CHE, the risk of infectious diseases outbreaks increases.^15^ Many factors contribute to the outbreak and transmission of such diseases, including: overcrowding, scarcity of safe water, poor sanitation and waste management, poor immunization levels and nutritional status, inadequate access to health care, in addition to the collapse of public health infrastructure hampering prevention and control programs. Since acute communicable diseases present an active major threat, the most common acute communicable diseases in CHE were chosen for this parameter; namely diarrheal illnesses, acute respiratory infections, measles, malaria, in addition to meningitis and hepatitis A. Naturally-emerging infectious diseases (like the Ebola virus outbreak in Zaire 1995) would be included, as would outbreaks due to biological weapons. In 2002, 207 outbreak events of international public health importance were verified, and 29% of them were recorded in countries affected by complex emergencies.^16^ As of 25 October 2010, the Haitian Ministry of Health reported 3,342 confirmed cases of cholera, with many suspected cases that are being investigated.^17^ In 1994, after the influx of 800,000 Rwandan refugees into North Kivu, Democratic Republic of the Congo, 43,000 deaths that were recorded in the first month were caused by diarrheal diseases; 60% of which were a result of cholera, and 40% were caused by shigella dysentery.^18^ Acute respiratory infections caused 63% of the morbidity in Nicaraguan refugees in Costa Rica in 1989.^18^

The estimated absolute number of cases with acute communicable disease cases is used in this parameter. In addition to this parameter, four out of the 12 parameters also utilize absolute numbers for affected persons (other than dead). These other parameters are: number of cases with traumatic/chemical/radiological injuries; number of children with acute malnutrition; number of displaced persons; and number of persons with inadequate living space. The baseline for these parameters is set at 20 so the cumulative total would add to a minimum of 100. This was purposefully designed since CRED lists “more than 100 affected persons” as one of its criteria to enter a disaster in its registry. For example, a CHE that causes 19 cases in each of these 5 parameters (a total of 95 affected persons) would get a score of 0 on each parameter, and would not qualify to enter the CRED registry.

**Table 3. Number of Cases with Acute Communicable Diseases:** 

Number of cases with acute communicable diseases	Severity score
<20	0
20-99	1
100-999	2
1,000-4,999	3
5,000-9,999	4
10,000-19,999	5
20,000-29,999	6
30,000-39,999	7
40,000-49,999	8
50,000-59,999	9
Greater than or equal to 60,000	10

**4. Number of Cases with Traumatic/Chemical/Radiological Injuries:** The absolute number of people with traumatic injuries attributable to CHE (blunt or penetrating, directly or indirectly) is another important indicator of the magnitude of emergencies. It adds another level of complexity facing the medical system and humanitarian response. In addition, chemical and radiological CHE also present an acute burden of disease requiring special capacities. The baseline of the scoring rubric in this parameter (<20 would get a zero) also stems from CRED criteria, as explained in parameter 3. The ceiling for this parameter is chosen to be 300,000 because the 2010 Haitian earthquake has also caused one of the highest numbers of documented acute injuries in the setting of CHE.^19^ Below is a table showing multiple examples (some CHE and others not), with their corresponding scores.^13^ ^19^ ^20^ ^21^

**Table 4. Number of Cases with Traumatic/Chemical/Radiological Injuries:** 

Number of cases with traumatic/chemical/radiological injuries	Severity score	Country, year, type	Number of cases
<20	0	None recorded by EM-DAT	N/A
20-99	1	Indonesia, 1928, earthquake	40
100-999	2	Sakhalin Island, 1927, earthquake	750
1,000-4,999	3	Vietnam, 2006, typhoon	2,000
5,000-9,999	4	Nepal, 1920, earthquake	6,553
10,000-19,999	5	Chile, 2010, 12,000	12,000
20,000-49,999	6	Iran, 1926, earthquake	30,000
50,000-99,999	7	Pakistan, 2008, earthquake	69,000
100,000-199,999	8	Japan, 1945, atomic bomb	110,000
200,000-299,999	9	India, 1984, chemical	200,000
Greater than or equal to 300,000	10	Haiti, 2010, earthquake	300,000

**5. Level of Health Care Services:** Evaluating the residual capacity of the healthcare system in the aftermath of a CHE is important to determine the scope of outside resources needed for a response. The 2004 Sphere Project^22^ described minimum standards required for healthcare provision in emergency settings, and was used as a template for the scoring of this parameter: (a) The first level of healthcare system is the community healthcare level. This level requires a minimum of one community health worker per 500-1,000 population; one skilled/traditional birth attendant per 2,000 persons; one supervisor per 10 home visitors; and one senior supervisor. (b) The second level of healthcare system is a peripheral health facility (for approximately 10,000 population) requiring a total of two to five staff with: minimum of one qualified health worker, based on one clinician per 50 consultations per day; and non-qualified staff for administering oral rehydration therapy (ORT), dressings, registration, administration, among other tasks. (c) The third level of healthcare system is a central health facility (for approximately 50,000 population) requiring a minimum of five qualified health workers; minimum of one doctor; one qualified health worker per 50 consultations per day (out-patient care); one qualified health worker per 20-30 beds, 24-hour services (in-patient care); one non-qualified health worker for administering oral rehydration therapy; one/two for pharmacy; one/two for dressings, injections, sterilization; one lab technician; and non-qualified staff for registration, security, among other tasks. (d) The fourth level of healthcare is a referral hospital with surgical capacity, with at least one doctor with surgical skills, and one nurse for 20-30 beds per shift.^22^

**Table 5. Levels of Health Care Services:** 

Levels of health care services	Severity score
All levels of heath care system are intact	0
Disrupted fourth level of health care; intact third, second and first levels	1
Disrupted fourth and third levels of health care; intact second and first levels	2
Disrupted second, third and fourth levels of healthcare; intact first level	3
All levels of health care disrupted	4

The 2010 Haiti earthquake, all levels of healthcare were disrupted in the acute phase, and hence this CHE would get a score of 4 at that time. Note that a retrospective evaluation of the pre-earthquake health system in Haiti using the above parameters indicates disrupted third and fourth levels of healthcare services.

**6. Number of Young Children (6-59 months) with Acute Malnutrition:** Malnutrition contributes to up to 55% of all children’s deaths in developing countries.^23^ ^24^ This percentage increases in acute emergency situations. Malnutrition and infections are intimately related; a malnourished child is more susceptible to disease, and a sick child is more likely to become malnourished. In order to measure the level of acute malnutrition in CHE, several methods are used, however, Weight for Height (WfH) for children between 6-59 months is recommended as the main indicator of malnutrition by most UN agencies, governments, and nongovernmental agencies. By convention, children with WfH of less than -2 standard deviations, or -2 Z scores below the median of reference (or edema), are considered acutely malnourished.^25^ ^26^ ^27^ ^28^ ^29^ ^30^ ^31^ The estimated absolute number of children with Z score of -2 or less (or with edema) is the basis of this parameter. This can be obtained by estimating the total number of children age 6-59 months affected, multiplied by the percentage of children who are categorized as acutely malnourished. Some regions in the world might have existing endemic malnutrition before the onset of CHE; however, this parameter would still require intervention whether acute or endemic. Evaluation of number of young children with acute malnutrition is also used routinely by various agencies and tools conducting assessments, which are included in this scale. The baseline of this parameter is chosen to be 20 and the remainder is stochastic. In the 2005 food crisis in Niger, MSF treated 63,000 children with acute malnutrition.^32^ This parameter would have a score of 10 under the proposed scoring rubric.

**Table 6. Number of Young Children with Acute Malnutrition:** 

Number of young children (age 6-59 months) with acute malnutrition	Severity score
<20	0
20-99	1
100-999	2
1,000-4,999	3
5,000-9,999	4
10,000-19,999	5
20,000-29,999	6
30,000-39,999	7
40,000-49,999	8
50,000-59,999	9
Greater than or equal to 60,000	10

**7. Number of Displaced Persons (internally displaced or refugees):** The displacement of people is a well recognized consequence of complex humanitarian emergencies. Displacement is either internal within countries (internally displaced person or IDP), or external across country borders referred to as “refugee” according to the Geneva Convention.^33^ According to the Internal Displacement Monitoring Centre (IDMC) , internally displaced persons are "persons or groups of persons who have been forced or obliged to flee or to leave their homes or places of habitual residence, in particular as a result of or in order to avoid the effects of armed conflict, situations of generalized violence, violations of human rights or natural or human-made disasters, and who have not crossed an internationally recognized State border."^34^ Those who are displaced need a set of complex of services including shelter, food, water, sanitation, healthcare, among others. The higher the absolute number of displaced, the more severe the humanitarian crisis is, and thus the more complex the needs and the response. This parameter includes both IDPs and refugees. The floor of this scoring rubric is set at 20, while the ceiling is set at 1,000,000. Several days after the Pakistan floods from the Indus River in August 2010, the UNICEF estimated that more than 3 million were in need of emergency assistance including shelter.^35^ This crisis would also translate to a score of 10 on this parameter.

**Table 7. Number of Displaced Persons:** 

Number of displaced persons	Severity score
<20	0
20-99	1
100-999	2
1,000-9,999	3
10,000-49,000	4
50,000-99,999	5
100,000-199,999	6
200,000-499,999	7
500,000-999,999	8
Greater than or equal to 1,000,000	9

**8. Number of Persons with Inadequate Living Space:** Shortage of space is a common problem in CHE, especially in temporary shelters for IDPs and refugees. Humanitarian assistance organizations should obtain, as quickly as possible, an estimate of the absolute number of people without adequate living space because of the risk of infectious outbreaks and violence. Early estimates are also necessary since providing temporary shelters require well-organized and time consuming logistical preparation measures. According to the Sphere Project, temporary planned or self-settled camps require a minimum surface area of 45 m^2^ per person, with a covered shelter area of not less than 3.5 m^2^ per person.^3^ The floor of this scoring rubric was set at 20. The number 200,000 was used as the ceiling since, according to Human Rights Watch, the Somali refugee camp in Dadaab, Kenya, is home to the largest population of refugees in the world, estimated at more than 200,000 refugees.^36^ In the aftermath of the Rwandan genocide in 1994, the Kibeho camp had a surface area of 9 km^2^ with up to 120,000 IDPs. In this scenario, the average surface area per person is about 75 m^2^, and the score on this parameter –for illustration purposes- would be zero.^37^ A more detailed survey within the camp may reveal a certain number with inadequate living space, and a higher score on this parameter.

**Table 8. Number of Persons with Inadequate Living Space:** 

Number of persons with surface area of less than 45 m^2^ or covered area of less than 3.5 m^2^	Severity score
<20	0
20-99	1
100-999	2
1,000-4,999	3
5,000-9,999	4
10,000-19,999	5
20,000-49,999	6
50,000-99,999	7
100,000-199,999	8
Greater than or equal to 200,000	9

**9. Water Quantity:** People can survive much longer without food than without water. In extreme situations, there may not be sufficient water available to meet basic needs, and in these cases supplying a survival level of safe drinking water is of critical importance. In addition, health problems are caused by poor hygiene due to insufficient water and by the consumption of contaminated water. Although water quantity is more important than water quality especially in the initial phase of CHE, quality is tightly linked to diarrheal diseases which are a major type of morbidity and mortality. The parameter of water quantity implies accessibility. Not only is it important to have available sources of water, but access to these sources is pivotal. The provision of water demands immediate attention from the start of a CHE. According to The Sphere Project 2011, the total basic water need is 7.5-15 liters per person per day (l/p/d).^3^ The minimum water quantity needed for survival is 2.5-3 l/p/d.^3^ Basic hygienic practices require 2-6 l/p/d, and basic cooking needs are 3-6 l/p/d.^3^ Based on the above guidelines for water requirements, a scoring rubric for water quantity is proposed in table 9. In 1994 in Rwanda, there were about 800,000 refugees, and the water availability for the first few weeks was only 0.2 liters/person/day. This would get a score of 9 on this parameter.^37^

**Table 9. Water Quantity:** 

Water quantity (liters/person/day)	Severity score
>15	0
12.5-15	1
10-12.4	2
7.5-9.9	3
6.5-7.4	4
5.5-6.4	5
4.5-5.4	6
3.5-4.4	7
2.5-3.4	8
<2.5	9

**10. Water Quality:** Testing water quality in the field may not be an easy task. It depends on the resources available to the assessment team. The most widely used tests detect and enumerate common fecal bacteria, such as fecal coliforms (mostly Escherichia coli). The presence of fecal coliform bacteria indicates that the water has been contaminated by feces of humans or other warm-blooded animals. Concentrations of fecal coliforms are usually expressed as the fecal coliform count per 100 milliliters (ml) of water. The OFDA guidelines categorize the water fecal coliform counts as reasonable quality if the count is 1-10 fecal coliforms/100 ml water, and polluted if more than 10 per 100 ml.^4^ Based on those guidelines, the following is the suggested scoring rubric for this parameter: The number of collected water samples depends on multiple factors including the number of water sources, accessibility to water sources, time limitation, and resources available (human and technical). More samples and better sampling techniques increase the accuracy of the water quality results. In 2009, a sample of 240 drinking water sources in Nyala City in South Darfur, Sudan, found 45.2% of sources contaminated with fecal coliforms. This CHE would get a score of 5 on this parameter.^38^

**Table 10. Water Quality:** 

Percentage of water samples with more than 10 fecal coliforms/100 ml	Severity score
0%	0
>0-9.9%	1
10-19.9%	2
20-29.9%	3
30-39.9%	4
40-49.9%	5
50-59.9%	6
60-69.9%	7
70-79.9%	8
80-89.9%	9
>90%	10

**11. Level of Sanitation Facilities:** Sanitation is a concern to all communities impacted by complex emergencies. The disruption and overcrowding of people accustomed to living in less crowded conditions in their own homes makes sanitation a critical issue. The minimum standards for available sewage systems or temporary sanitation facilities are considered the basis for the scoring rubric in this parameter, based on The Sphere Project guidelines.^3^

**Table 11. Levels of Sanitation Facilities:** 

Levels of sanitation facilities	Severity score
Intact well developed sanitary system for disposal of excreta	0
Minor disruption of a well developed sanitary system for disposal of excreta	1
Two or more toilets (or latrines) per 20 people, or two or more family latrines per 4 families, or two or more trench latrines per 100 people	2
One toilet (or latrine) per 20 people, or one family latrine per 4 families, or one trench latrine per 100 people	3
Less than one toilet (or latrine) per 20 people, or one family latrine per 4 families, or one trench latrine per 100 people	4
Complete absence of any system for disposal of excreta	5

**12. Gender-based violence:** Attacks against women are the most common type of gender-based violence and is particularly disruptive to the movement of the population, the maintenance of agrarian sustenance farming, and security within the home. The Convention on the Elimination of All Forms of Discrimination against Women (CEDAW), adopted in 1979 by the UN General Assembly, is often described as an international bill of rights for women defining what constitutes discrimination against women and proposing an agenda to end discrimination. In 1992, CEDAW defined gender-based violence as “violence that is directed against a woman because she is a woman or that affects women disproportionately. It includes acts that inflict physical, mental or sexual harm or suffering, threats of such acts, coercion and other deprivations of liberty.”^39^ Below is a proposed stochastic scoring rubric for this public health parameter in CHE, as proposed by the authors. Examples of systematic gender based violence include former Yugoslavia where the European Community estimates 20,000-50,000 Bosnian women were systematically raped as a means for ethnic displacement.^40^ ^41^ This CHE would get the maximum score of 4 on this parameter, taking the upper estimate, on the basis of sexual harm alone.

**Table 12. Gender-Based Violence:** 

Gender-based violence	Severity score
None	0
Isolated single events (not daily)	1
Average occurrences approximately one daily	2
Average occurrences approximately multiple daily	3
Systematic gender-based violence	4

## Discussion

Assessment of public health impact in complex humanitarian emergencies is a daunting task of unparalleled importance.^42^ ^43^ ^44^ ^45^ ^46^ It is the guiding compass for the type and scale of response undertaken by various stakeholders. The objectives of assessment –especially in the acute phase- are to measure the magnitude of the complex emergency and population size affected; identify the vulnerable population groups with high risk of death or disease; determine the present health priorities and potential public health problems; and gather information on the availability of food, water and shelter, clinics and hospitals.^47^ Having background information about the affected population, such as demographics and baseline health indicators (e.g. CMR), is of prime importance to the overall assessment process. Coordination among various organizations and entities performing assessment is also vital for both consistency and efficiency. The Johns Hopkins and Red Cross Red Crescent Public Health Guide in Emergencies highlights some important considerations including, but not limited to, defining the terms of reference and the objectives of the assessment, determining the priorities to be considered, and selecting how, in what order and what timeline the information will be gathered.^49^The guide encourages the use of standardized checklists and measures, as proposed by the PHISS model.

The proposed PHISS, illustrated above, constitute quantitative measures for the assessment of the public health impact of Complex Humanitarian Emergencies. Conceptually, it may be used as a “big picture” multi-sectoral quantitative assessment tool, by providing objective scores on specific parameters. It can potentially be used in entirety or partially, both cross-sectionally or longitudinally across time. The cumulative PHISS score ranges from a minimum of 0 to a maximum of 100. It uses absolute numbers instead of percentages (e.g. excess mortality instead of mortality rates), in order to depict the actual scale of the impact that would be applicable to affected populations of various sizes. Whenever possible, existing scoring rubric criteria were utilized for the parameters. This scale does not undermine the value of descriptive methods, nor is it meant to replace existing guidelines and tools, but rather to build upon and augment them. Adopting and applying such a quantitative scale in CHE maybe useful for guiding response efforts, longitudinal assessment within each CHE, and comparative studies between various CHE. Such methodology would compliment current descriptive assessment tools. This approach may streamline the post-CHE assessment phase by providing an overall quantitative objective measure, and provide the opportunity of pooling comparable data from various sources and organizations. The opportunity to aggregate data from various agencies and organizations increases the precision of assessment data, and optimizes the speed and accuracy of resource allocation in CHE. In longitudinal assessments, comparable periods of time need to be taken into consideration. A uniform quantitative assessment method would help facilitate inter-agency communication and reduce subjective variations and errors in assessment. The scale may allow the standardization of the various approaches currently in use across health organizations, which would reduce redundancy of assessment efforts. Moreover, applying an overall quantitative score to different events allows for historical comparisons between disparate CHE. Of note, the reason PHISS has not been applied to one single historical CHE, is that data on the 12 proposed parameters are not readily available and do not reside in one or multitude of data sources.

It is of prime importance to note that sound and standardized sampling methodology, in addition to uniformity of measurement techniques, are key to the accuracy of the PHISS results. Technical issues regarding cluster sampling and data aggregation for various parameters require thorough evaluation and further recommendation.

## Limitations and Strengths

There are several limitations and challenges to our proposed scale; most are related to the availability of baseline population figures, and to the accuracy and standardized methodology of measuring the 12 proposed parameters. First, some of those parameters (e.g. excess mortality) require baseline population figures which may be lacking in CHE. This potential problem is not unique to this proposed tool, and may be remedied by estimation methods. Second, there would still be some variation in the data collected by various assessors. A training program for potential assessment teams can minimize this variation. Third, PHISS is not meant to be an exhaustive tool, and some parameters might be suggested to be added to the scale (e.g. food availability by calories). Other CHEs might have special characteristics that are not included. Yet, the parameters included in PHISS constitute the major public health categories in the acute phase of CHE, as identified by many benchmark guidelines including The Sphere Project. Fourth, there is an inherent element of stochasticity -though chosen by consensus- when assigning scoring rubrics for some of the parameters. In addition, the maximum score for few parameters varies, giving more weight to some more than others. These restrictions were due to the absence of standardized reference ranges in the literature. However, every attempt was made to use available data and references when applicable. Fifth, the sum of all the parameters' scores might not always reflect the complexity of the CHE. Sixth, we comparing different CHE, the period of time need to be standardized for parameters one and tow (excess deaths and under-5 excess deaths). Finally, one parameter, namely water quality, requires some basic technological capacity. Simple commercial kits are readily available on the market, can be used to collect the water quality data. Of note, pooling data from existing tools may readily provide quantitative scores on certain PHISS parameters (e.g. estimated number of excess deaths). Since testing the validity and feasibility of PHISS was not the objective of this proposed study, the authors hope that the introduction of this quantitative scale would invigorate scientific discussion and prompt organizations and agencies with sufficient resources to apply PHISS prospectively in future CHE.

## Conclusion

Measuring the public health impact of complex humanitarian emergencies is germane to objectively and accurately identify the scope and scale of the critical needs and relief priorities for affected populations. A new quantitative assessment tool (PHISS) has been proposed, based on The Sphere Project “minimum standards”, in order to streamline and standardize the post-CHE assessment phase. PHISS is a conceptual disaster metric that has not yet been validated, and studies are needed with prospective data collection to test its validity, feasibility and reliability.
